# Engineering aldolases as biocatalysts^☆^

**DOI:** 10.1016/j.cbpa.2013.12.010

**Published:** 2014-04

**Authors:** Claire L Windle, Marion Müller, Adam Nelson, Alan Berry

**Affiliations:** 1 Astbury Centre for Structural Molecular Biology, University of Leeds, Leeds LS2 9JT, UK; 2 School of Molecular and Cellular Biology, University of Leeds, Leeds LS2 9JT, UK; 3 School of Chemistry, University of Leeds, Leeds LS2 9JT, UK

## Abstract

Aldolases are seen as an attractive route to the production of biologically important compounds due to their ability to form carbon–carbon bonds. However, for many industrial reactions there are no naturally occurring enzymes, and so many different engineering approaches have been used to address this problem. Engineering methods have been used to alter the stability, substrate specificity and stereospecificity of aldolases to produce excellent enzymes for biocatalytic processes. Recently greater understanding of the aldolase mechanism has allowed many successes with both rational engineering approaches and computational design of aldolases. Rational engineering approaches have produced desired enzymes quickly and efficiently while combination of computational design with laboratory methods has created enzymes with activity approaching that of natural enzymes.

## Highlights

• Aldolase enzymes are attractive candidates as biocatalysts.
• Stability, stereoselectivity and substrate specificity of aldolases have been altered.
• Aldolases with desirable activities have been produced using a variety of methods.
• Combining computational with other methods produces efficient designer aldolases.

**Current Opinion in Chemical Biology** 2014, **19**:25–33

This review comes from a themed issue on **Biocatalysis and biotransformation**

Edited by **Jeffrey C Moore** and **Uwe T Bornscheuer**

For a complete overview see the Issue and the Editorial

Available online 4th January 2014

1367-5931/$ – see front matter, © 2013 The Authors. Published by Elsevier Ltd. All rights reserved.

**http://dx.doi.org/10.1016/j.cbpa.2013.12.010**

## Introduction

The formation of carbon-carbon bonds is central to organic chemistry and the aldol condensation [1–4], the reaction of two carbonyl compounds to generate a new β-hydroxyl carbonyl compound, is an important tool in building up complexity of organic molecules, since up to two new stereogenic centres are made during the formation of the new C–C bond. Aldol structural units are found in many naturally occurring molecules and are the result of reactions catalysed by the aldolase family of enzymes. These enzymes convert their substrates into the aldol products in high yield with high specificity under mild conditions, but also with great control over the relative and absolute configurations of the new stereogenic centres created. These properties make aldolase-catalysed routes attractive for the production of biologically significant compounds, as these tend to contain multiple functional groups and are often water-soluble making conventional synthetic routes more difficult [5]. However, naturally occurring aldolases do not exist for many industrially important reactions and protein engineering, directed evolution and *de novo* enzyme design [6–10] have all been used to alter properties such as stability, substrate specificity and stereoselectivity to produce tailor made aldolases for use as biocatalysts. Since we reviewed this area in 2008 [11] it is pleasing to see an increasing use of protein engineering to manipulate aldolases as new biocatalysts, both in their own right and as part of chemical cascade reactions leading to important products (see Table 1 for a summary of recent examples of engineering aldolases). Additionally, the growing area of computational enzyme design [12] has shown the way towards *de novo* designed aldolases [13], especially when combined with directed evolution to improve the activity of the ‘designer’ aldolases [14^••^].

## Engineering of aldolases for enhanced stability and activity

Enzymes have many desirable features as biocatalysts, but denaturation at higher temperatures, intolerance towards organic solvents and the possibility of substrate inhibition are drawbacks which may limit their use in industrial environments or enzymatic cascade reactions. However, these problems may be overcome by engineering. For example, the thermostability and solvent tolerance of fructose-1,6-bisphosphate aldolase (FBP-aldolase) was increased using family DNA shuffling [15] of the *fda* genes from *Escherichia coli* and *Edwardsiella ictaluri* and a fourth generation variant was identified which displayed an average 280-fold higher half-life at 53 °C than either parent. The same variant also displayed enhanced activity in various polar and non-polar organic solvents — directed evolution in this case providing beneficial properties over and above those that were screened for.

Aldolases have also been engineered towards enhanced activity at lower temperature as this may be more beneficial in an industrial setting. A random library, generated by error-prone PCR, of the hyperthermophilic 2-keto-3-deoxygluconate aldolase (KDG-aldolase) from *Sulfolobus acidocaldarius* which has an optimal activity for the condensation of d,l-glyceraldehyde with pyruvate at 95 °C, was screened for enhanced activity at 50 °C. The V193A variant has threefold higher activity than the wild-type enzyme, with the highest increase in activity at 40 °C for both the natural aldehyde acceptor, as well as a number of unnatural acceptor aldehydes. Interestingly, this mutation had little influence on the thermostability of the enzyme as the observed *t*_1/2_ at 90 °C was similar to that of the parent aldolase [16]. This decoupling of activity and stability demonstrates the potential for optimizing extremely thermostable aldolases for synthesis reactions at moderate temperatures.

The engineering of aldolases towards enhanced activity at different temperatures may help to make them applicable for use in cascade reactions, where combinations of thermophilic and mesophilic enzymes may require their optimal temperatures to be matched. In addition, increased activity may also be needed to generate useful enzymes for cascade reactions. For example the rate-limiting enzyme in the bioconversion of xylose to ethanol in *Pichia stipites* is a transaldolase and directed evolution was used to create a transaldolase with increased activity in converting sedoheptulose 7-phosphate (S7P) and glyceraldehyde 3-phosphate (G3P) into fructose 6-phosphate (F6P) and erythrose 4-phosphate (E4P) and therefore increase the production of ethanol, a conversion that is of great interest to industry as it may lead to renewable fuels [17]. An error prone PCR strategy was used and two variants were identified, Q263R and K190 M, with >5-fold increases in *k*_cat_/*K*_M_ *in vitro*. The Q263R mutant was introduced into *P. stipitis* using homologous recombination and ethanol production in the resulting strain was shown to be 36% higher than with the parental strain [18^••^].

Another example of the beneficial engineering of an aldolase for use in cascade reactions involves 2-deoxy-ribose-5-phosphate aldolase (DERA). This enzyme has been applied as a biocatalyst for the synthesis of (3*R*,5*S*)-6-chloro-2,4,6-trideoxyhexapyranoside, a valuable chiral precursor for statin drugs such as atorvastatin (Lipitor). (3*R*,5*S*)-6-chloro-2,4,6-trideoxyhexapyranoside can be formed from chloroacetaldehyde (CAA) and two equivalents of acetaldehyde in a sequential tandem enzymic aldol reaction (Table 1); however, economically efficient large-scale synthesis was hampered by the enzyme's low affinity for CAA and the concentrations of CAA needed for efficient biocatalysis lead to rapid and permanent enzyme inactivation. Error prone PCR and DNA recombination were used to engineer DERA for increased stability to CAA, and a number of variants resistant to inhibition at CAA concentrations up to 400 mM CAA were identified (e.g. variant M185V or variants altered at the C-terminus). In addition, variants with increased activity were also identified by error-prone PCR, for example variant F200I, which showed 14-fold improved activity and a twofold to threefold lower *K*_M_ for CAA. Subsequent combination of the F200I mutation with the ΔY259 C-terminal deletion or with a variant containing Y259T and a 9-residue extension to the C-terminus resulted in ∼10-fold higher catalytic activity in the presence of 1 M acetaldehyde and 500 mM CAA than the wild-type under industrially relevant conditions [19].

## Engineering aldolases with varied substrate specificities

Enzymes have high specificity, but the narrow substrate range is problematic if no natural enzyme exists for a desired, specific reaction. There are many examples where protein engineering has been applied to aldolases to broaden or change the substrate specificities, for both the aldehyde acceptor and the ketone donor, and to exploit catalytic promiscuity for the production of synthetically useful compounds.

The Class I pyruvate-dependent 2-keto-3-deoxy-6-phosphogluconate-aldolase (KDPGA) catalyses the cleavage of 2-keto-3-deoxy-6-phosphogluconate (KDPG) into pyruvate and glyceraldehyde 3-phosphate and has been the subject of many studies to alter its substrate specificity [20^••^,21–24]. Recent engineering has used both directed evolution [21] and structure-based mutagenesis [20^••^] to expand its substrate range to non-functionalized electrophilic substrates and pyridine carboxaldehyde substrates, respectively. Furthermore, the activity of the variant KDPGA with the pyridine carboxaldehyde substrate (4*S*)-2-keto-4-hydroxy-4-(2′-pyridyl) butyrate (*S*-KHPB) maintains high stereoselectivity at a similar rate to that of the wild-type enzyme with KDPG. These new substrate specificities could prove useful in the synthesis of important antifungal and antimicrobial compounds.

In general, aldolases are much more specific for their aldol donor substrate than for their acceptor. This has proved problematic, especially with the dihydroxyacetone phosphate (DHAP) dependant aldolases, where DHAP is expensive, relatively unstable and where the phosphate group is often not required in the final product. There have been a number of attempts to redesign these enzymes to use the non-phosphorylated donor, dihydroxyacetone (DHA), by using directed evolution [25] or rational methods using point mutations to redesign the phosphate binding pocket [26^•^]. In this respect fructose-6-phosphate aldolase (FSA) is of great interest as it has been shown to utilize multiple donor substrates such as dihydroxyacetone (DHA), hydroxyacetone and hydroxybutanone [27]. FSA also provides a route to the production of iminocyclitols which are attractive drug candidates [28]. FSA has been the subject of many studies to alter its substrate specificity for different acceptor aldehydes and to increase its affinity for the specific donor DHA [29^•^,30]. Another enzyme that uses DHA rather than DHAP is transaldolase (Tal) and, interestingly, FSA activity has been conferred on this enzyme by replacement of a single phenylalanine by tyrosine (F178Y) in the active site [31]. This F178Y variant has also been the subject of further study to increase its activity with non-phosphorylated acceptor aldehydes. Structure-guided mutagenesis identified residues in the phosphate binding pocket that, when mutated, prevent phosphorylated acceptors from binding. This has produced an enzyme that can synthesize polyhydroxylated, non-phosphorylated compounds and be used in enzymatic cascade synthesis of this type of compound [32].

Many enzymes have been shown to have catalytic promiscuity and as well as using engineering to subvert the substrate specificity of natural aldolases, attempts are now being made to enhance the catalytic promiscuity of other enzyme classes to produce novel aldolases. An early example of the conversion of one enzyme activity into another type of reaction was the conversion of an alanine racemase into an aldolase by a single active site point mutation [33]. This variant enzyme catalysed a reaction similar to threonine aldolase with rates and specificities comparable with the native enzyme. More recently 4-oxalocrotonate tautomerase (4-OT) was shown to be promiscuous in having low aldolase activity towards the condensation of acetaldehyde and benzaldehyde to yield cinnamaldehyde. This low activity has been enhanced by a single point mutation, F50A, which increased the *k*_cat_/*K*_M_ for the aldolase activity by 600-fold compared to that of the wild-type [34^•^].

Lipases have also been reported to display promiscuous aldolase activity [35,36] and recently asymmetric aldol reactions between acetone and 4-nitrobenzaldehyde (catalysed by porcine pancreas lipase) [37] and aromatic and heteroaromatic aldehydes with cyclic ketones (catalysed by chymopapain, nuclease p1, alkaline protease BLAP and acidic protease AUAP) [38,39] have been described. Care must, however, be taken since some of these apparent promiscuous activities have been shown to be due to a combination of the natural activity of the enzyme coupled with spontaneous chemical reaction with the other aldol partner to generate the misleading promiscuous activity (reviewed in [40]). Promiscuous aldolase activity has also been found for macrophomate synthase which catalyses the addition of the enolate of pyruvate (generated on the enzyme by decarboxylation of oxaloacetate) with a wide range of structurally complex aldehydes to yield 3-deoxysugars [41]. This system has advantages over known natural pyruvate-dependent aldolases as it has a broad substrate spectrum.

## Engineering of aldolases for increased stereospecificity

The biological outcomes of the interactions of stereoisomers of small drug molecules with their targets can be dramatically different and the global market for enantiomerically pure, active pharmaceutical ingredients (APIs) is increasing rapidly. However, the chemical synthesis of enantiomerically pure compounds can be challenging, and most often relies on the classical resolution of a racemate. Harnessing enzymes as chiral catalysts is viable in both the small scale and industrial synthesis of enantiomerically enriched compounds. In this respect, protein engineering of enzymes to enhance or alter the stereochemical outcome of an enzyme reaction is of great importance and much attention has been focused on aldolases, as up to two stereo-centres may be generated during the carbon-carbon bond forming step [11]. In recent years there has been much progress in using many engineering methods ranging from directed evolution [42] to rational redesign [43^••^,44,45] to produce products with high stereochemical control. Improved biocatalysts have also been found by screening available environmental DNA libraries. In this way, a natural variant of DERA was discovered that produced (3*R*,5*S*)-6-chloro-2,4,6-trideoxyhexapyranoside (see above), with a diastereomeric excess of 99.8% and an enantiomeric excess of 99.9% [46]. This variant also had a higher tolerance to the inhibiting substrate chloroacetaldehyde and was more efficient than the *E. coli* variant, allowing lower quantities of the enzyme to be used in the process. Both these factors increase the commercial and industrial viability of the biocatalytic process.

The ability to engineer or evolve the stereochemical outcome of an aldolase reaction was first demonstrated for tagatose-1,6-bisphosphate aldolase [47] and *N*-acetylneuraminic acid lyase [42]. More recently, rational redesign has been carried out on the Class II aldolase BphI to switch the stereochemical outcome of the reaction of pyruvate with acetaldehyde. First, the substrate specificity of BphI was changed to favour propionaldehyde over acetaldehyde [48] using site-directed mutagenesis based on modelling of the structure using the orthologous enzyme DmpG [49]. While wild-type BphI is highly selective for *si* face attack of the pyruvate enolate onto the aldehyde carbonyl to produce 4(*S*)-hydroxy-2-oxopentanoate as product, two variants (Y290F and Y290S) utilize *both* the 4(*S*)-enantiomer and the 4(*R*)-enantiomer [48]. It is suggested that the hydroxyl group of Tyr290 sterically restricts the binding of the 4(*R*) enantiomer and its removal permits both isomers to bind. Further modelling suggested that Leu87 interacts with the C4-methyl of 4(*S*)-hydroxy-2-oxoacids. Double mutants at positions 87 and 290 were constructed and the stereochemical outcome of the reaction was found to be switched from 4*S* in the wild-type to 4*R* in the L87N/Y290F and L87W/Y290F double mutants [43^••^].

**Figure 1:**
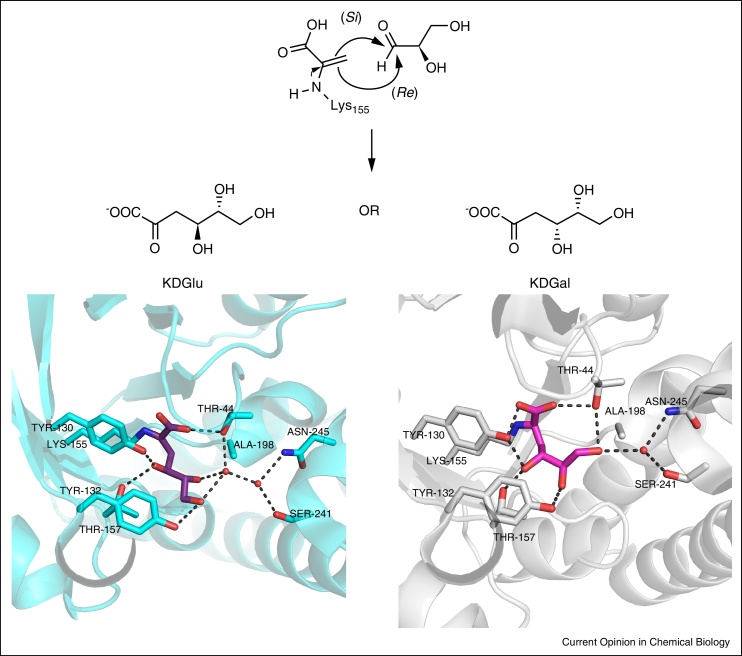
The top panel shows the attack of the lysine bound enamine form of pyruvate onto the either the *Si* or *Re* face of d-glyceraldehyde, to produce either KDGlu or KDGal. The bottom panel shows the binding interactions of KDGlu (on the left) and KDGal (on the right) in the active site of KDGA. Differences in the stabilization of C5-OH and C6-OH of KDGlu and KDGal can be seen. The C5-OH residue of d-KDGal was shown to hydrogen bond directly with Tyr-132 whereas the C5-OH residue of d-KDGlu makes water mediated hydrogen-bonding interactions within the active site. There are also differences in the bonding of the terminal C6-OH residue; in d-KDGal it interacts directly with Thr-44 whereas in d-KDGlu it is directly stabilized by hydrogen bonding with Tyr-132 and water mediated hydrogen bonding.

Another example of engineering of stereochemistry involves the enzyme 2-keto-3-deoxygluconate aldolase (KDGA). This enzyme has broad substrate specificity but poor diastereo-control for the reaction of pyruvate, either with the natural substrate d-glyceraldehyde (where a 55:45 mixture of d-2-keto-3-deoxy-gluconate (d-KDGlu): d-2-keto-3-deoxy-galactonate (d-KDGal) is produced) or with the enantiomer of the substrate, l-glyceraldehyde. However stereoselectivity could be engineered into this reaction by conformationally locking the substrate as either the d-glyceraldehyde acetonide or the (*S*)-enantiomer [50]. To achieve the same goal by engineering the enzyme, detailed X-ray crystallographic analysis of the structures of both d-KDGlu and d-KDGal bound to KDGA was used [51] to identify residues for mutation to generate a pair of complementary stereoselective enzymes [44]. Interest was focused on the differences in binding the C5 and C6 hydroxyl groups of d-KDGlu and d-KDGal and the epimeric C4-OH group of both diastereoisomers and lead to saturation mutagenesis of Thr-157 (Figure 1) and combination with mutations at Tyr132. Two double mutants, T157C/Y132V and T157F/Y132V, were found which catalysed the stereoselective formation of KDGlu in an improved ∼92%dr. To create the complementary KDGal-specific enzyme, a double mutant T157V/A198L was identified from structural information that would disrupt the hydrogen bonding network for KDGlu and this enzyme resulted in the production of KDGal with an improved 72%dr. Study of the enzyme structure suggested that the binding of KDGal could be further improved by adding a third mutation (at Asp-181) to create the T157V/A198L/D181Q triple mutant, which indeed showed that the dr for the formation of KDGal could be improved to 88%dr. This work [44] demonstrated again that stereochemically complementary variants can be produced from a stereochemically promiscuous enzyme, but also highlighted the power of structurally informed mutagenesis in the construction of new aldolases as biocatalysts.

## Computational design of aldolases

**Figure 2:**
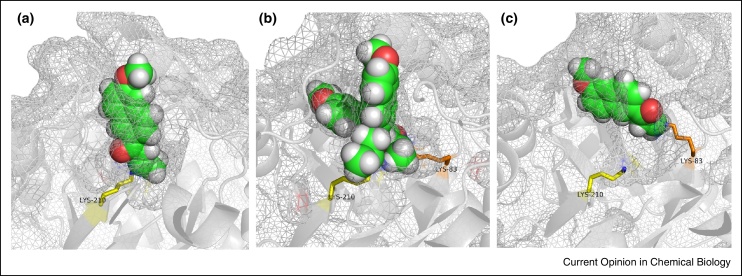
The remodelling of the active site of the designer retroaldolase RA95.0 [14^••^]. **(a)** The structure of RA95.0 (PDB code 4A29) showing the designed catalytic lysine residue, Lys210 (yellow) and the 1, 3 diketone mechanism-based inhibitor (green). **(b)** The structure of variant RA95.5 (PDB code 4A2S) showing the two different binding modes of inhibitor, one using Lys210 (yellow) and the other, the newly introduced Lys83 (orange). **(c)** The structure of RA95.5-5 (PDB code 4A2R) showing that the active site location has switched during evolution to the new lysine Lys83, and that Lys210 is not modified in RA95.5-5.

Much progress has been made towards altering existing enzymes for tailored, stereochemically controlled aldol condensations using a combination of protein engineering strategies. An increasingly common feature of such experiments is the combination of engineering and/or directed evolution with structural modelling, and computational strategies [7,12]. An ultimate example is the *de novo* computational design of an aldolase [13]. Although the starting activities of such designed enzymes is low, random mutagenesis at the active site and at more distant locations can be used to improve the activity [52^••^]. To explore the structural basis of these changes and to augment the activity of the designer aldolase, further rounds of directed evolution were carried out and X-ray crystal structures of the enzyme in complex with a mechanism-based inhibitor were solved after each stage of evolution. In the initial designer enzyme (RA95.0) the inhibitor reacts covalently with Lys210 as was intended for the designer enzyme. However, during the evolution of increased activity (variant RA95.5) a new lysine was introduced into the active site (Lys83) during cassette mutagenesis and unexpectedly RA95.5 is modified twice by the mechanism-based inhibitor — once at Lys210, as in RA95.0, and once at the newly introduced Lys83 (Figure 2). After further rounds of error prone PCR variant RA95.5-5 was constructed which contained additional mutations and which was >20-fold more efficient than RA95.5 and >1700-fold more active than the original *in silico* design. Structurally this variant showed further modulation of loops of the protein, but interestingly was only modified by the inhibitor at Lys83, implying that this new binding site is more evolvable than the original designer site. Subjecting this evolved retro-aldolase to further error prone PCR produced an enzyme with activity approaching that of a natural aldolase, notable for an artificial enzyme. This work demonstrates how powerful the combination of computational and traditional methods can be and also allows insight into the mechanisms that lead to enhanced catalytic efficiency [52^••^].

## Conclusions and future perspectives

The synthetic utility of aldolase enzymes may be substantially increased using protein engineering approaches. Complementary approaches have been exploited to improve the properties of aldolases including their stability, substrate scope and stereoselectivity. Excitingly, the increased understanding of the function of aldolase variants, together with computational approaches, can help focus protein engineering experiments on specific, functionally important residues. Such approaches can improve the efficiency of searching within sequence space, enabling more rapid discovery of enzymes with the required synthetically valuable properties. The future use of these important enzymes looks bright with the ability to link engineered aldolases with other enzymes in novel constructed pathways and organisms opening the way to their increased use in synthetic biology to more easily produce valuable but useful complex compounds.

## References and recommended reading

Papers of particular interest, published within the review period, have been highlighted as:

• of special interest

•• of outstanding interest

## Figures and Tables

**Table 1:** A selection of recent examples of engineering of aldolases for use as biocatalysts

Enzyme	Reactions	Ref.
2-Deoxyribose-5-phosphate aldolase	Natural	[19]
	

	Engineered	
		

2-Keto-3-deoxy-6-phosphogluconate aldolase	Natural	[20^••^,21]
	

	Engineered	
		

Rhamnulose-1-phosphate aldolase	Natural	[26^•^]
	

	Engineered	
		

Fructose-6-phosphate aldolase	Natural	[29^•^,30]
	

	Engineered	
		

Transaldolase	Natural	[31,32]
	

	Engineered	
		

4-Oxocrotonate tautomerase	Natural	[34^•^]
	

	Engineered	
		

BphI	Natural	[42,47]
	

	Engineered	
		

2-Keto-3-deoxygluconate aldolase	Natural	[43^••^]
	

	Engineered	[43^••^]
		

	Engineered increased stability at lower temperatures	
